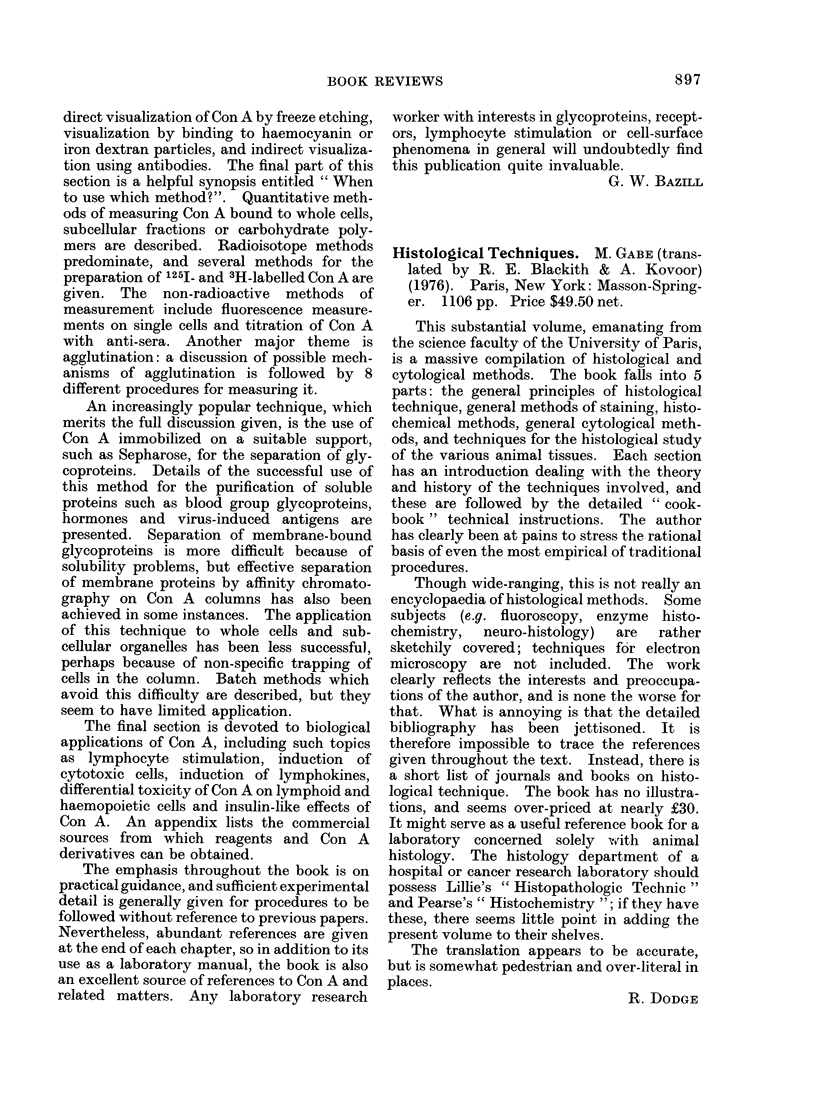# Histological Techniques

**Published:** 1977-06

**Authors:** R. Dodge


**Histological Techniques.** M. Gabe (translated by R. E. Blackith & A. Kovoor) (1976). Paris, New York: Masson-Springer. 1106 pp. Price $49.50 net.

This substantial volume, emanating from the science faculty of the University of Paris, is a massive compilation of histological and cytological methods. The book falls into 5 parts: the general principles of histological technique, general methods of staining, histochemical methods, general cytological methods, and techniques for the histological study of the various animal tissues. Each section has an introduction dealing with the theory and history of the techniques involved, and these are followed by the detailed " cookbook " technical instructions. The author has clearly been at pains to stress the rational basis of even the most empirical of traditional procedures.

Though wide-ranging, this is not really an encyclopaedia of histological methods. Some subjects (*e.g.* fluoroscopy, enzyme histochemistry, neuro-histology) are rather sketchily covered; techniques for electron microscopy are not included. The work clearly reflects the interests and preoccupations of the author, and is none the worse for that. What is annoying is that the detailed bibliography has been jettisoned. It is therefore impossible to trace the references given throughout the text. Instead, there is a short list of journals and books on histological technique. The book has no illustrations, and seems over-priced at nearly £30. It might serve as a useful reference book for a laboratory concerned solely with animal histology. The histology department of a hospital or cancer research laboratory should possess Lillie's " Histopathologic Technic " and Pearse's " Histochemistry "; if they have these, there seems little point in adding the present volume to their shelves.

The translation appears to be accurate, but is somewhat pedestrian and over-literal in places.

R. Dodge